# Mapping of EFO terms from the GWAS catalog data to multiple ontologies at the Rat Genome Database

**DOI:** 10.1093/database/baag008

**Published:** 2026-02-27

**Authors:** Stanley J F Laulederkind, G T Hayman, Shur-Jen Wang, Carissa A Park, Mary L Kaldunski, Mahima Vedi, Marek A Tutaj, Logan Lamers, Jennifer R Smith, Jeffrey L de Pons, Melinda R Dwinell, Anne E Kwitek

**Affiliations:** Rat Genome Database, Department of Physiology, Medical College of Wisconsin, 8701 Watertown Plank Rd, Milwaukee, WI 53226, USA; Rat Genome Database, Department of Physiology, Medical College of Wisconsin, 8701 Watertown Plank Rd, Milwaukee, WI 53226, USA; Rat Genome Database, Department of Physiology, Medical College of Wisconsin, 8701 Watertown Plank Rd, Milwaukee, WI 53226, USA; Rat Genome Database, Department of Physiology, Medical College of Wisconsin, 8701 Watertown Plank Rd, Milwaukee, WI 53226, USA; Department of Animal Science, Iowa State University, 2255 Kildee Hall, 806 Stange Road, Ames, IA 50011-3150, USA; Rat Genome Database, Department of Physiology, Medical College of Wisconsin, 8701 Watertown Plank Rd, Milwaukee, WI 53226, USA; Rat Genome Database, Department of Physiology, Medical College of Wisconsin, 8701 Watertown Plank Rd, Milwaukee, WI 53226, USA; Rat Genome Database, Department of Physiology, Medical College of Wisconsin, 8701 Watertown Plank Rd, Milwaukee, WI 53226, USA; Rat Genome Database, Department of Physiology, Medical College of Wisconsin, 8701 Watertown Plank Rd, Milwaukee, WI 53226, USA; Rat Genome Database, Department of Physiology, Medical College of Wisconsin, 8701 Watertown Plank Rd, Milwaukee, WI 53226, USA; Rat Genome Database, Department of Physiology, Medical College of Wisconsin, 8701 Watertown Plank Rd, Milwaukee, WI 53226, USA; Rat Genome Database, Department of Physiology, Medical College of Wisconsin, 8701 Watertown Plank Rd, Milwaukee, WI 53226, USA; Rat Genome Database, Department of Physiology, Medical College of Wisconsin, 8701 Watertown Plank Rd, Milwaukee, WI 53226, USA

## Abstract

The laboratory rat, *Rattus norvegicus*, is an important model of many human diseases, and experimental findings in the rat have relevance to both human physiology and disease. The Rat Genome Database (RGD, https://rgd.mcw.edu/) is a model organism database that provides access to a wide variety of curated rat data including disease associations, phenotypes, pathways, molecular functions, biological processes, cellular components, and chemical interactions for genes, quantitative trait loci (QTL), and strains. Because the laboratory rat is used often as a model of human physiology and disease, RGD has incorporated data from the NHGRI–EBI (National Human Genome Research Institute-European Bioinformatics Institute) catalog of human genome-wide association studies (GWAS) (https://www.ebi.ac.uk/gwas/). This provides the basis for easy integration of RGD data for rat and other species with human SNP (single nucleotide polymorphism)-phenotype associations. When data from different sources use different vocabularies, there must be some standard way to compare the data. Since the human GWAS data are annotated with Experimental Factor Ontology (EFO) terms, RGD needed to map those EFO terms to various ontologies used at RGD for annotating rat genomic and strain data, so the human data can be translationally associated with the wealth of preclinical data including rat genes, rat QTL, and rat strains. To bridge the ontology/vocabulary gap, the curators at RGD have mapped all the EFO terms (http://www.ebi.ac.uk/efo) used to annotate the human GWAS SNPs in the NHGRI–EBI Catalog of human GWAS Catalog Data (https://www.ebi.ac.uk/gwas/) to multiple ontologies used at RGD. RGD has used the mappings to translate human GWAS disease/phenotype EFO annotations into RGD Disease Ontology annotations, human phenotype ontology annotations, clinical measurement ontology annotations, and vertebrate trait ontology annotations. Also, RGD has made the ontological mappings available via Simple Standard for Sharing Ontological Mappings (SSSOM) files on the RGD download site (https://download.rgd.mcw.edu/ontology/mappings/).

## Introduction

Since 1850, *Rattus norvegicus* (the laboratory rat) has been the model organism of choice for many investigations into the mechanisms of the genetic and environmental factors that affect human disease [1,2]. Nearly 2 million published research articles describing rat models of human physiology and disease reflect the extensive use of the rat in laboratories around the world. Since the completion of the rat genome sequence in 2004 [3], >40 inbred rat strains commonly used as disease models have been sequenced and genomic variations among these strains identified, providing valuable tools for linking genotypes to phenotypes [4]. Continued advancements in genetic-modification technologies have led to the generation of more refined models, further contributing to the increasing popularity of the rat as a genetic model of disease [5]. Because of the value of existing and emerging rat datasets, the Rat Genome Database (RGD, https://rgd.mcw.edu) was created in 1999 and has evolved into the leading resource for rat genomic, genetic, phenotype, and strain data. The main responsibilities of RGD are to collect and display rat data and provide software tools for access and analysis of the data. This collected and validated information is imported for use at several large data resources, such as Ensembl, NCBI, and UniProtKB. RGD provides official nomenclature for rat genes, quantitative trait loci (QTLs), strains, and markers, as well as unique identifiers for each of these. Current emphasis by publishers and funding agencies on the public availability of data underscores the need for interoperability of data, terminology, and identifiers.

There are many ontologies/vocabularies (https://bioportal.bioontology.org/) that exist to describe biomedical data in a controlled, formatted, and coded manner. One of the main advantages of describing data using ontologies is that they allow automated reading and reasoning of the data (https://www.ontotext.com) by computer. Many of these ontologies/vocabularies overlap because of species-specific efforts, domain specificity, disagreements on creation or maintenance methods, and other user-based reasons. Therefore, to achieve interoperability in biomedical science data, there must be a mapping system between ontologies to indicate identity or similarity between data from different sources. A method commonly used to link terms in different ontologies/vocabularies is the cross reference (XREF), usually represented by an ID (CURIE/compact URI), so that similar or identical terms from different ontologies can be mapped to each other. Ontology mapping can be provided by a service of the European Bioinformatics Institute (https://www.ebi.ac.uk/spot/oxo/) using existing sources, or it can be done independently by way of automated and/or manual methods. Regardless of how the ontology term matching is accomplished, it is useful to the community of biomedical ontology users to document ontology term matches using a standard like SSSOM (Simple Standard for Sharing Ontological Mappings) [6]. Data integration and interoperability between different data sources depend on standards for mapping between different representations of the same or similar objects.

The Experimental Factor Ontology (EFO) [7] (https://www.ebi.ac.uk/efo) is a cross-domain ontology designed for gene expression data (specifically gene Expression Atlas data, https://www.ebi.ac.uk/gxa/home). The EFO integrates many different types of data in one ontology/controlled vocabulary. Therefore, to integrate data [such as human genome-wide association study (GWAS)] annotated with EFO terms, mapping of EFO terms to many different ontologies is necessary. Qualitative phenotype data, quantitative phenotype data, and disease data at RGD are currently curated with 9 different ontologies/vocabularies (Table 1).

**Table 1:** Ontologies used at RGD for curation of phenotype and disease data

Data type at RGD	Ontology used at RGD
Rat phenotype (qualitative)	Mammalian Phenotype ontology (MP)
Rat phenotype (quantitative)	Vertebrate Trait ontology (VT)
	Clinical Measurement Ontology (CMO)
	Measurement Method Ontology (MMO)
	Experimental Condition Ontology (XCO)
	Uberon ontology (UBERON)
	Cell ontology (CL)
Human phenotype (qualitative)	Human Phenotype Ontology (HPO)
Disease (qualitative)	RGD Disease Ontology (RDO) (axiomatized and extended version of the human Disease Ontology (DO)

RGD curation of physiological and pathological data for genes, QTLs, and rat strains is linked to human, mouse, and other model organism data by many different controlled vocabularies/ontologies. Since phenotype and disease data at RGD are described using MP (Mammalian Phenotype Ontology, for rat and mouse) [8], VT (Vertebrate Trait Ontology, for rat) [9], CMO (the Clinical Measurement Ontology, for rat) [10], RDO (RGD Disease Ontology, for human, rat, mouse, and other species) [11], and HPO (Human Phenotype Ontology, for human) [12], EFO [7] terms needed to be linked to all these ontologies so that the GWAS data would be more integrated and interoperable with the existing phenotype and disease data at RGD. Therefore, RGD has mapped the whole EFO [7] (https://www.ebi.ac.uk/efo) subset of terms found in the GWAS Catalog Data to the following vocabularies/ontologies: RDO [RGD’s axiomatized and expanded version of the Disease Ontology (DO)] (https://diseaseontology.org/do) [11], HPO, MP, CMO, and VT. To extend mapping in phenotype, RGD has downloaded HPO–MP mappings from the Monarch project (https://github.com/mappingcommons/mh_mapping_initiative/blob/master/mappings/mp_hp_mgi_all.sssom.tsv) [13] and assigned XREFs accordingly.

## Import of human GWAS data into RGD

Because the laboratory rat is often used as a model of human physiology and disease, RGD decided to import SNPs (single nucleotide polymorphisms)and their associated traits from the NHGRI–EBI (National Human Genome Research Institute-European Bioinformatics Institute) Catalog of human GWAS (https://www.ebi.ac.uk/gwas/). This provides the basis for easy integration of RGD data for rat and other species with human SNP phenotype associations. Since the human GWAS data are annotated with EFO terms, RGD needed to map those EFO terms to various ontologies used at RGD for annotating rat genomic and strain data, so the human data can be translationally associated with the wealth of preclinical data from model organisms, including rat genetic, genomic, and strain data.

As of September 2025, the total number of datasets in the human GWAS catalog (https://www.ebi.ac.uk/gwas/) was greater than 115 000. Each submitted/accepted study must contain data describing at least 100 000 SNPs. Each SNP represents at least one phenotype/trait. A SNP may also have a ‘background trait’ associated with it. All these traits are described using > 9000 EFO terms (Table 2).

**Table 2:** Tally of EFO terms associated with imported GWAS records and number of EFO terms mapped to relevant ontologies at RGD

EFO term subsets	Total subset numbers as of April 2025		
Total # of unique EFO terms associated with imported GWAS records	7601		
Total # of unique EFO/HP/MONDO hybrid terms associated with imported GWAS records	2009		
		**Automated matches**	**Manual matches**
# of EFO terms mapped to RDO/DO	1930	1630	300
# of EFO terms mapped to HPO	518	347	171
# of EFO terms mapped to CMO	469	77	392
# of EFO terms mapped to MP	209	139	70
# of EFO terms mapped to VT	2052	82	1970

## Automated term mapping

EFO-target ontology mapping has been done with both automated and manual approaches. As a first step in the mapping, an automated matching algorithm (https://github.com/rat-genome-database/ontology-loadpipeline/blob/master/src/main/java/edu/mcw/rgd/dataload/ontologies/test/TermNameMatcher.java) compared the GWAS-associated EFO terms to RDO/DO terms, HPO terms, MP terms, CMO terms, and VT terms. The algorithm first tokenized the EFO term and its synonyms, which essentially means that the EFO terms/synonyms were broken down into single words and normalized [converted to all lowercase letters with separators (spaces or punctuation marks) discarded]. The tokens were then alphabetized and concatenated into a ‘keyword’ used to search ‘keywords’ generated by the same tokenization of each target ontology. After tokenization and normalization of EFO terms/synonyms and target ontology terms/synonyms, the EFO ‘search-keyword’ was compared against all ‘keywords’ from the target ontology. Matches were tallied (no match equals 0, one match equals 1, etc.) to make a prioritized list of matches that curators could check. In addition to the keyword matching, EFO XREF IDs were compared to XREFs in the target ontology to find exact matches present between EFO terms and terms in the targeted ontology. The automated matches were validated by curators before bulk loading of synonyms (exact, broad, narrow, or related—determined by curator) and XREFs from EFO was done in the RGD instances of RDO/DO, HPO, MP, and to the official version of CMO (at RGD).

For the EFO mapping to disease and phenotype ontologies, successful automated matching of terms was between 67% and 85%. That was relatively high compared to the Clinical Measurement and VT ontologies. The high degree of matching to DO was not surprising, because many EFO terms have Medical Subject Headings (MeSH), MONDO, and DO XREFs. Many disease terms across the different vocabularies and ontologies are connected either by entry term, synonym, or XREF. The EFO mapping to CMO and VT was completely different with automated matching only accounting for 16% and 4%, respectively, for CMO and VT. The lower automated matching numbers for these last two ontologies may reflect the fact that that CMO has been developed mainly to describe experimental rat data and VT has been developed to describe mouse, rat, and livestock traits. Since this mapping project involves a subset of EFO terms taken from a human GWAS dataset, one might expect fewer crossover of terms, synonyms, and XREFs between EFO and the more animal-based ontologies of CMO and VT. Partly because of this, it took more manual effort to assign mapping between EFO and CMO/VT.

## Manual term mapping

The Manual term mapping process was a two-step process involving one curator and one curator/editor. The first curator was tasked with determining matches by a combination of personal knowledge of the relevant biomedical area and by searching the target ontology with the EFO term. The closeness of the match would be defined by the assigned synonym: exact, broad, narrow, or related. Following the decision by the first curator, the second curator/editor would determine, by essentially the same process as the first curator, whether to accept, reject, or change the mapping of the EFO term and associated EFO accession ID.

Each EFO-target ontology set was done with 3–5 curators and one curator/editor. After all the curator lists were compiled and edited, the mappings were bulk loaded into the RGD instances of the ontologies (mappings to VT were loaded at Iowa State University).

## EFO–RDO mapping

As of September 2025, there were >3750 EFO disease terms in the human GWAS catalog. Since the human DO [14] (https://disease-ontology.org/do) is the standard DO/vocabulary used by the Alliance for Genome Resources [15] (https://www.alliancegenome.org/) and by hundreds of biomedical researchers worldwide, RGD needed to map EFO disease terms to RDO/DO so that human GWAS data could be linked to disease data from rat, mouse, human, and other species in RGD. One reason for RGD using an axiomatized and extended version of DO lies in the difference between human disease and animal models of disease. There are disease terms available in the MeSH vocabulary (https://meshb.nlm.nih.gov/) that contain the word ‘experimental’ which are not in DO. Use of these terms allows RGD to be more accurate than applying a human disease term to a rat disease or other model organism disease. Beyond the species-specific challenges of using the standard DO, there are gaps and deficiencies in DO that exist because it is still a growing and developing ontology. For these reasons, RGD has developed RDO [11] and has used RDO for many years.

The first step in mapping EFO to RDO was done by an automated matching algorithm that compared text strings of terms and synonyms, together with database XREF IDs between EFO terms and RDO terms. The automated, algorithmically matched terms (1630) were confirmed by curator review. The manually matched terms (300) were identified solely by curator review.

EFO terms that matched by automated algorithm or manual assessment were linked to RDO/DO terms by adding the EFO accession IDs as XREFs and EFO terms as ‘exact’ (having the same meaning), ‘narrow’ (representing a subset), ‘broad’ (associated with more than one term), or ‘related’ (closely associated term, but not fully interchangeable with target term) synonyms to the RGD RDO/DO term entries. The type of synonym was determined by curator review.

## EFO–HPO mapping

HPO [12] has been used for 10 years at RGD [16] to annotate human genes and QTLs. The human GWAS loci, defined by an associated SNP variant, were designated as GWAS_QTL in RGD, because they represent a specific type of phenotypic locus, different from traditional QTLs. Both disease terms and physiological phenotype terms from EFO are relevant to terms in HPO. Some EFO terms are imported from HPO, so some terms in EFO are inherently linked to HPO. To guarantee EFO to HPO coverage for the bulk of the imported GWAS data, RGD has mapped EFO terms associated with GWAS variants to HPO terms. This will provide connections between human genes and older human QTLs in RGD to the data from the imported human GWAS catalog.

The first step in mapping EFO to HPO was done by an automated matching algorithm that compared text strings of terms and synonyms, database XREFs, etc. between EFO terms and HPO terms, like the algorithm used for EFO–RDO/DO mapping. The automated, algorithmically matched terms (347) were confirmed by curator review. The manually matched terms (171) were not identified automatically, but solely by curator review.

EFO terms that matched by automated algorithm or manual assessment were linked to HPO terms in the RGD term browser (https://rgd.mcw.edu/rgdweb/ontology/search.html), similar to the process for EFO–RDO/DO mapping.

## EFO–MP mapping

Rat genes, QTLs, and strains have been annotated with MP ontology terms for 20 years at RGD [8]. Created mainly to annotate mouse variants generated by chemical mutagenesis and genetic engineering [8], MP is also useful for annotating any type of genetic variants in rat. Since both rat and mouse serve as model organisms for human diseases and phenotypes, it is important to connect human variant data to model organism variant data. Mapping EFO terms used for human GWAS annotation to MP terms is another way to bolster the translational connection of data between human and model organisms.

The first step in mapping EFO to MP was done by an automated matching algorithm that compared text strings of terms and synonyms, database XREFs, etc. between EFO terms and MP terms, like the algorithm used for EFO–RDO mapping. The automated, algorithmically matched terms (139) were confirmed by curator review. The manually matched terms (70) were not identified automatically, but solely by curator review.

EFO terms that matched by automated algorithm or manual assessment were linked to MP terms as was done with EFO–HPO mapping.

## EFO–CMO mapping

The CMO is an ontology developed at RGD in support of a quantitative phenotype data curation effort [14]. That quantitative phenotype data curation populates a part of RGD called the PhenoMiner database [17]. It encompasses two large, imported datasets: PGA data (>30 000 records) [18] and NBRP data (>3000 records) [19], about 550 studies (31 000 records) from extensive curation of the biomedical literature by RGD curators, and phenotype data directly uploaded by researchers (http://rgd.mcw.edu/wg/home/phenominer-data-upload) [20] (RGD:153 344 611, RGD:38 548 922, 8818 records).

The original goal of PhenoMiner was to be able to compare rat strains based on quantitative phenotype data. With the import of the large dataset from the human GWAS Catalog (https://www.ebi.ac.uk/gwas/) annotated with EFO terms, it was clear that a connection between the rat PhenoMiner data and the human GWAS data would be beneficial to database users looking for rat models of human phenotype and disease.

The first step in mapping EFO to CMO was again done by an automated matching algorithm that compared text strings of terms and synonyms, and database XREF IDs between EFO terms and CMO terms, like the algorithm used for EFO–RDO mapping. The matched terms (77 CMO terms) were confirmed by curator review. Another 392 EFO terms were mapped to CMO terms by manual review of EFO terms that were left unmatched by automated algorithm (Table 2).

## EFO–VT mapping

The VT ontology is an ontology/vocabulary designed to aid in the comparison of data within and between species and to facilitate investigation of the genetic basis of traits [21]. It began as a collaboration among the database teams at the Animal QTL Database (QTLdb, https://www.animalgenome.org/QTLdb), Mouse Genome Informatics/Mouse Phenome Database (MGI/MPD, https://www.informatics.jax.org/;https://phenome.jax.org/), and the RGD (https://rgd.mcw.edu/). The premise of VT fits well with the purpose of comparing human GWAS data to rat QTL and strain data. By mapping the EFO terms that are associated with human GWAS QTLs to VT terms associated with rat QTLs and rat strains, comparison between human phenotype and rat phenotype data is made easier. Comparisons to other species annotated with VT, such as livestock species in the QTLdb, are also made possible with the EFO–VT mapping.

The EFO–VT mapping was done by the same type of automated/manual match combination as described above for the other term matching. The final approval of EFOVT mappings (XREFs and synonyms) was done by the Bioinformatics Team at Iowa State University, the home of the VT ontology. 2052 of the EFO terms/IDs associated with human GWAS variants have been evaluated and mapped to VT terms and assigned as synonyms and/or XREFs. Nearly all these mappings were manually curated.

## GWAS annotation translation

**Figure 1:**
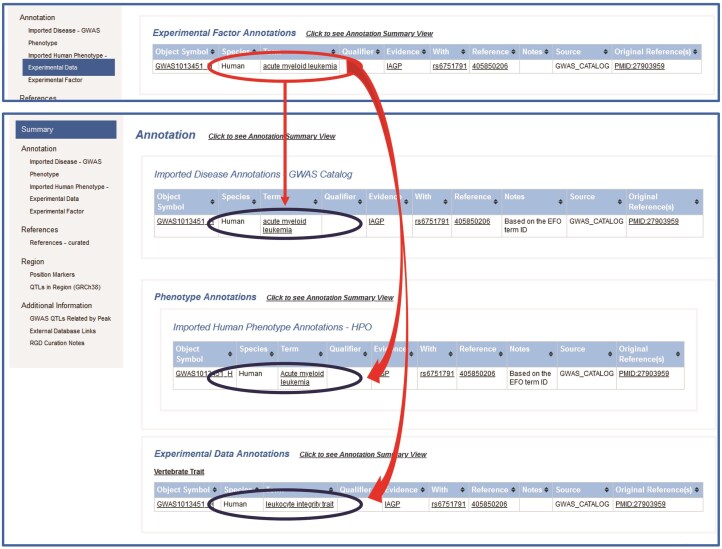
Based on EFO/RDO, EFO/HPO, and EFO/VT mapping, RDO, HPO, and VT annotations are translated from the EFO annotation and assigned to the human SNP QTL GWAS1013451_H.

All human GWAS results (>600 000 SNPs) were downloaded from the NHGRI–EBI GWAS Catalog to generate >500 000 GWAS SNP QTLs in RGD. To make it easier for database users to search for the SNPs and to compare phenotype/disease annotations to rat data, the EFO/HPO, EFO/RDO, and EFO/VT mappings were leveraged to assign corresponding annotations to GWAS QTLs with matching EFO terms/IDs (Fig. 1).

**Figure 2:**
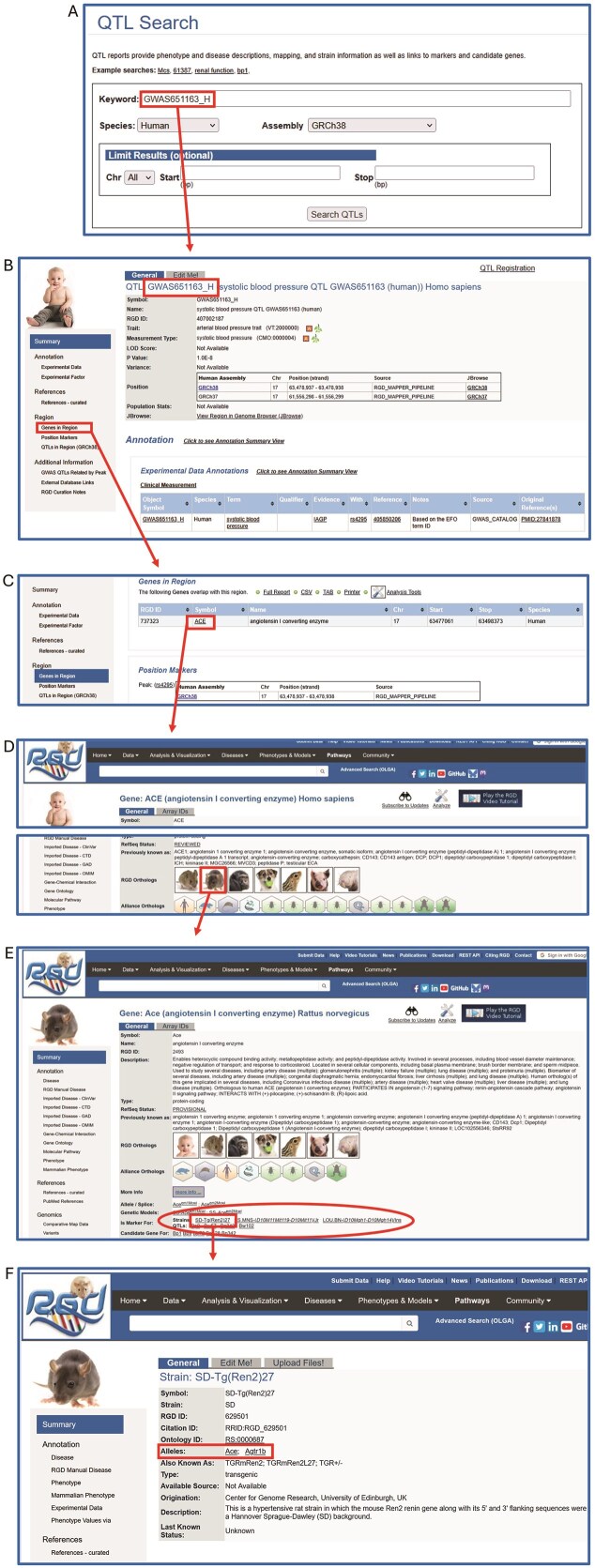
Path from human QTL GWAS651163_Hpage to a list of rat strains (models for hypertension) and QTLs. (A) QTL search; (B) Human QTL report page with disease annotation and relevant genomic data; (C) ‘Genes in Region’ of the human QTL page, showing ACE, the gene in which the human QTL GWAS651163_Hresides; (D) Human ACE report page with link to rat Ace report page; (E) Rat Ace page with links to model strains and QTLs; ‘hypertension’; (F) SD-Tg(Ren2)27 strain page with related alleles of Ace and Agtr1b.

The connections between human QTLs and related rat data can be exploited in a variety of ways. One way is to download annotation sets from the RGD download site (https://download.rgd.mcw.edu) and analyze with whatever bioinformatic tools a researcher may have. RGD also hosts many analysis tools through which data can be discovered and manipulated. One such example of using the RGD website to find rat strains relevant to a human GWAS QTL is shown in Fig. 2. The following is the sequence of steps followed to generate the example illustrated in Fig. 2:

1. Take the QTL of interest (GWAS651163_H) and search for it on the QTL search page (Fig. 2A) or the general search on any RGD page.
2. The result returned is the human GWAS651163_Hpage (Fig. 2B), which includes the annotation ‘systolic blood pressure’).
3. Scroll down the human QTL page to ‘Genes in Region’ (Fig. 2C).
4. Click on ‘ACE’ (circled in red) to link to the human gene report page for ACE/angiotensin I converting enzyme, followed by linking over to the rat ortholog page for Ace (Fig. 2D).
5. On the rat Ace gene report page (2E) strain models and QTLs relevant to hypertension and blood pressure are listed in the ‘Is Marker For’ section (red oval).
6. Clicking on ‘SD-Tg(Ren2)27’ (red rectangle) will lead to the strain report page which describes this rat strain which is a model for hypertension (Fig. 2F).
7. Gene variants (including an Ace allele) associated with this strain are listed in the ‘Alleles’ section of the page (red rectangle).

Starting with a human GWAS QTL, a user can use a series of manipulations on the RGD website to find both a relevant rat gene (>13 000 genes are currently linked to the human GWAS QTLs via orthology and related annotations) and a list of rat strains (1725 strains are currently linked to the human GWAS QTLs via related annotations and associated gene variants) that could be used to model diseases or conditions related to the original human QTL of interest.

## Future plans

To keep up with additional EFO terms from ongoing import of human GWAS records, a quality control algorithm will monitor incoming records from the NHGRI–EBI GWAS catalog and create a log of all EFO terms that are new to RGD. The logs will be monitored by curators who will create synonyms and/or XREFs in the appropriate ontologies in RGD.

To expand the interrelatedness of data in RGD, additional ontology term mapping will be pursued. Some of the next work will involve disease-phenotype mapping between RDO and MP, RDO and HPO, and disease-trait mapping between RDO and VT.

## Summary

The RGD has recently imported a large dataset (∼115 000 studies/>500 000 SNPs/phenotype associations) from the NHGRI–EBI Catalog of human GWAS (https://www.ebi.ac.uk/gwas/). Since those GWAS loci are annotated with EFO terms, and since no other annotations at RGD use EFO, RGD needed to map EFO to various other ontologies, to make the GWAS data interoperable with existing RGD data. Mapping of EFO terms to RDO/DO, HPO, MP, CMO, and VT was done by a combination of automated and manual methods, and SSSOM files of the ontology term mappings have been made available on the RGD download site. Annotations were transferred from the EFO annotations attached to the SNPs to RDO, HPO, and VT annotations via the EFO/RDO, EFO/HPO, and EFO/VT mappings. A monitoring system will be put in place to keep the mappings updated, based on new GWAS data brought in by the established pipeline.
